# Comparative analysis of kVCT- and MVCT-guided helical tomotherapy for total body irradiation: evaluation of process times and residual setup errors

**DOI:** 10.1093/jrr/rraf062

**Published:** 2025-10-07

**Authors:** Yuta Omi, Ryuichi Yada, Tatsuya Hasegawa, Ken Shishido, Keita Sakai, Tomotaka Kinoshita, Katsumasa Nakamura, Yoshiyuki Itoh, Arisa Takeuchi

**Affiliations:** Department of Radiation Oncology, Anjo Kosei Hospital, 28 Higashi-Hirokute, Anjo-cho, Anjo City, Aichi 446-8602, Japan; Department of Regional Medical Management Studies, Hamamatsu University School of Medicine, Handayama 1-20-1, Chuo-ku, Hamamatsu City, Shizuoka 431-3192, Japan; Department of Radiation Oncology, Anjo Kosei Hospital, 28 Higashi-Hirokute, Anjo-cho, Anjo City, Aichi 446-8602, Japan; Department of Radiological Technology, Anjo Kosei Hospital, 28 Higashi-Hirokute, Anjo-cho, Anjo City, Aichi 446-8602, Japan; Department of Radiological Technology, Anjo Kosei Hospital, 28 Higashi-Hirokute, Anjo-cho, Anjo City, Aichi 446-8602, Japan; Department of Radiological Technology, Anjo Kosei Hospital, 28 Higashi-Hirokute, Anjo-cho, Anjo City, Aichi 446-8602, Japan; Department of Radiation Oncology, Hamamatsu University School of Medicine, Handayama 1-20-1, Chuo-ku, Hamamatsu City, Shizuoka 431-3192, Japan; Department of Radiation Oncology, Anjo Kosei Hospital, 28 Higashi-Hirokute, Anjo-cho, Anjo City, Aichi 446-8602, Japan; Department of Radiation Oncology, Anjo Kosei Hospital, 28 Higashi-Hirokute, Anjo-cho, Anjo City, Aichi 446-8602, Japan

**Keywords:** total body irradiation (TBI), tomotherapy, megavoltage computed tomography (MVCT), kilovoltage computed tomography (kVCT)

## Abstract

Helical tomotherapy-based total body irradiation (TBI) traditionally employs megavoltage computed tomography (MVCT) for image-guided setup; however, its 390 mm field of view (FOV) and long acquisition times constrain workflow efficiency and whole-body alignment. This study evaluated whether a newly implemented whole-body fan-beam kilovoltage CT (kVCT; 500 mm FOV) can streamline this process. In a retrospective study involving 14 patients treated with a Radixact X9 system (September 2021–September 2023), we timed the patient setup, imaging, registration, re-setup, and beam delivery for each upper-body (UB) and lower-body (LB) segment. Residual setup errors were measured along the lateral, longitudinal, and vertical axes. The kVCT shortened the initial setup cycle (setup + imaging + registration) from 25.4 ± 4.6 to 15.9 ± 3.3 min for UB and from 14.5 ± 3.8 to 9.4 ± 2.4 min for LB (*P* < 0.001 for both). The total fraction time, including delivery time, decreased from 71.8 ± 7.5 to 56.7 ± 5.3 min. When residual errors exceeded 5 mm, the additional time required for a second cycle was nearly halved with kVCT (7.3 vs. 14.3 min for UB; 4.8 vs. 8.2 min for LB). The kVCT maintained mean absolute residual errors below 2 mm in all axes, and every 95th-percentile value remained within the 5 mm tolerance recommended for tomotherapy-based TBI. These time savings are expected to reduce intrafraction motion and staff workload. Overall, whole-body kVCT enables faster, comprehensive image guidance while preserving accuracy, thereby streamlining tomotherapy-based TBI and reducing the burden on patients and clinical staff.

## INTRODUCTION

Total body irradiation (TBI) is extensively used as a preparatory procedure for hematopoietic stem cell transplantation [1, 2]. When combined with chemotherapy, TBI plays a pivotal role in eliminating malignant cells and serves as an immunosuppressive agent for preventing graft rejection [1, 2]. Conventional TBI is administered using two opposing fields with an extended source-to-skin distance [1, 3], which restricts dose optimization in the organs at risk [4–8].

Tomotherapy, which is characterized by helical irradiation facilitated by gantry rotation and couch movements, has been recognized as a promising alternative for administering TBI [9], and several studies have demonstrated its effectiveness [3, 10, 11], particularly in enabling lung-sparing TBI while ensuring that the targeted dose is maintained within the rib cage [12, 13].

Helical computed tomography (CT) scanners are an integral component of tomotherapy systems [9]. Helical fan-beam kilovoltage (kV) CT, which has recently been introduced in tomotherapy, represents a significant advancement, expanding the field of view (FOV) from 390 to 500 mm and offering enhanced image quality and substantially reduced scan times compared with megavoltage (MV) and cone-beam CT [14–16]. Yang *et al.* reported that compared with MVCT, kVCT significantly reduces scanning and registration times in head, thorax, and pelvis protocols, thus enhancing treatment throughput and efficiency [17].

However, the clinical utility of kVCT has not yet been evaluated in the context of TBI. Compared with localized treatment, TBI presents unique challenges: extended craniocaudal scan ranges, longer treatment times, and an increased risk of intrafraction motion. The conventional MVCT-based workflow in TBI often involves multiple partial-body scans to limit the imaging time [10]. This segmented approach narrows the evaluation range and complicates assessment, particularly when anatomical changes occur outside the imaged regions. With its extended FOV and faster acquisition, whole-body kVCT may overcome these limitations by enabling a single comprehensive scan.

To the best of our knowledge, this is the first study to quantitatively compare three image-guidance strategies for helical tomotherapy-based TBI: (1) partial-body MVCT, as currently practiced in clinical workflows; (2) whole-body kVCT, as newly implemented in our institution; and (3) a simulated whole-body MVCT protocol designed to match the craniocaudal coverage of kVCT. By isolating the impact of the scan method and coverage extent, this comparison provides practical data for optimizing setup workflows in tomotherapy-based TBI.

This study aimed to compare the efficiency and setup accuracy of kVCT- and MVCT-guided helical tomotherapy for TBI. Specifically, we evaluated the time required for patient setup, image acquisition, image registration, re-setup, and delivery, as well as the residual pre-treatment setup errors for critical anatomical regions.

## MATERIALS AND METHODS

### Patient selection

This retrospective study involved 14 patients who underwent TBI at a tomotherapy facility between September 2021 and September 2023. All patients were treated using the Radixact X9 system (Accuray Inc., Sunnyvale, CA, USA). Each patient received treatment in two segments: upper body (UB) and lower body (LB). Table 1 summarizes the prescribed doses and the imaging modalities used for patient setup verification. Since its introduction at our institution in April 2022, kVCT has been used as the default modality for setup verification in TBI. In this study, patients from ID 8 onward were treated using kVCT.

**Table 1:** Prescribed dose and imaging modality used for patient setup verification

Patient ID	Prescription	Modality			
(Tx order)		1fr	2fr	3fr	4fr
1	12 Gy/4fr	M	M	M	M
2	12 Gy/4fr	M	M	M	M
3	4 Gy/2fr	M	M		
4	4 Gy/2fr	M	M		
5	12 Gy/4fr	M	M	M	M
6	4 Gy/2fr	M	M		
7	4 Gy/2fr	k	k		
8	12 Gy/4fr	k	k	M^a^	k
9	4 Gy/2fr	k	k		
10	4 Gy/2fr	k	k		
11	4 Gy/2fr	M^a^	k		
12	4 Gy/2fr	k	k		
13	12 Gy/4fr	k	k	k	k
14	12 Gy/4fr	k	k	k	k

Symbols: Tx, Treatment; M, megavoltage computed tomography; k, kilovoltage computed tomography

^a^kVCT was unavailable owing to a system error

### Immobilization and planning CT

**Figure 1:**
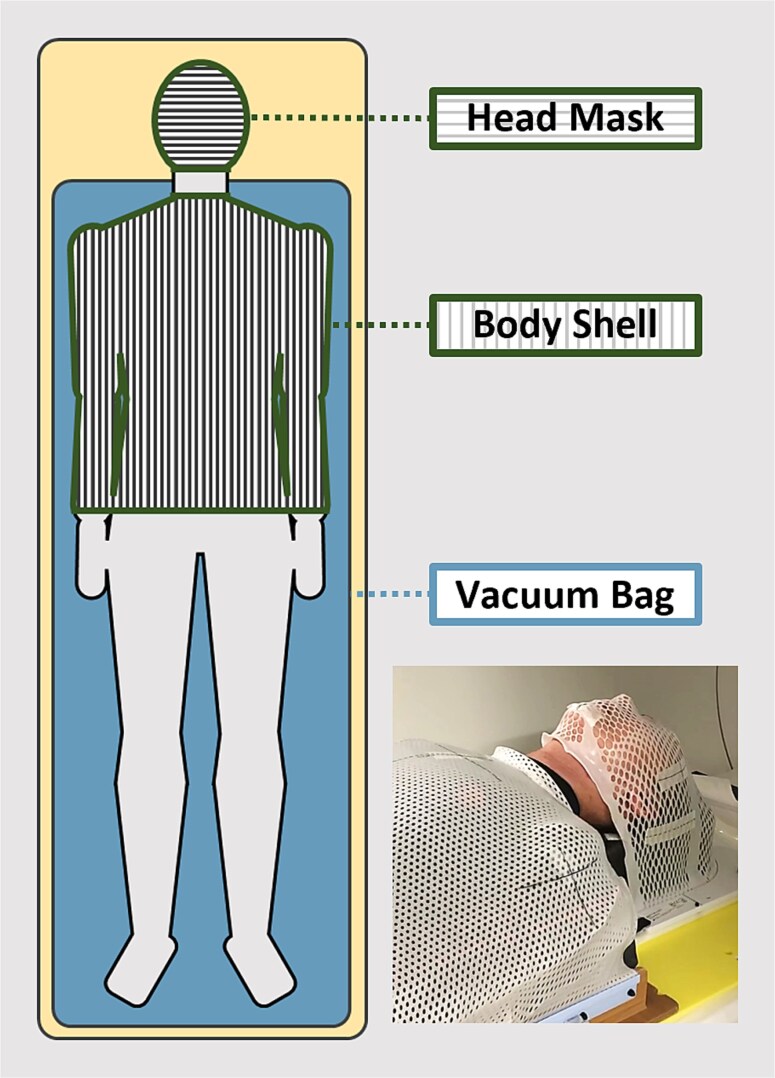
Patient immobilization.

The patient immobilization process is illustrated in Fig. 1. The patients were positioned supine on a vacuum cushion and secured with masks to stabilize the head and body. Imaging was performed using a wide-bore CT scanner. Owing to the limited range of table movement, both head- and feet-first CT scans were performed to ensure complete whole-body coverage.

### Patient setup verification

**Figure 2:**
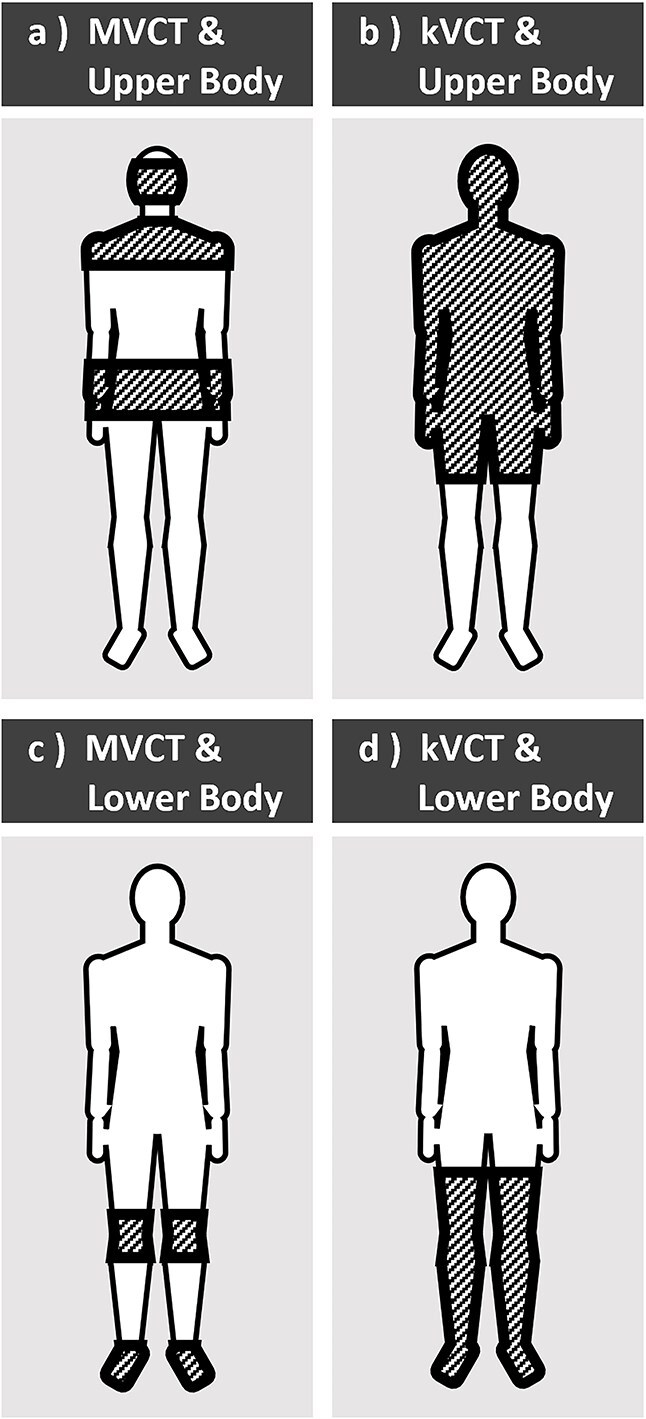
Image acquisition range. Hatched regions indicate the image acquisition range.

**Figure 3:**
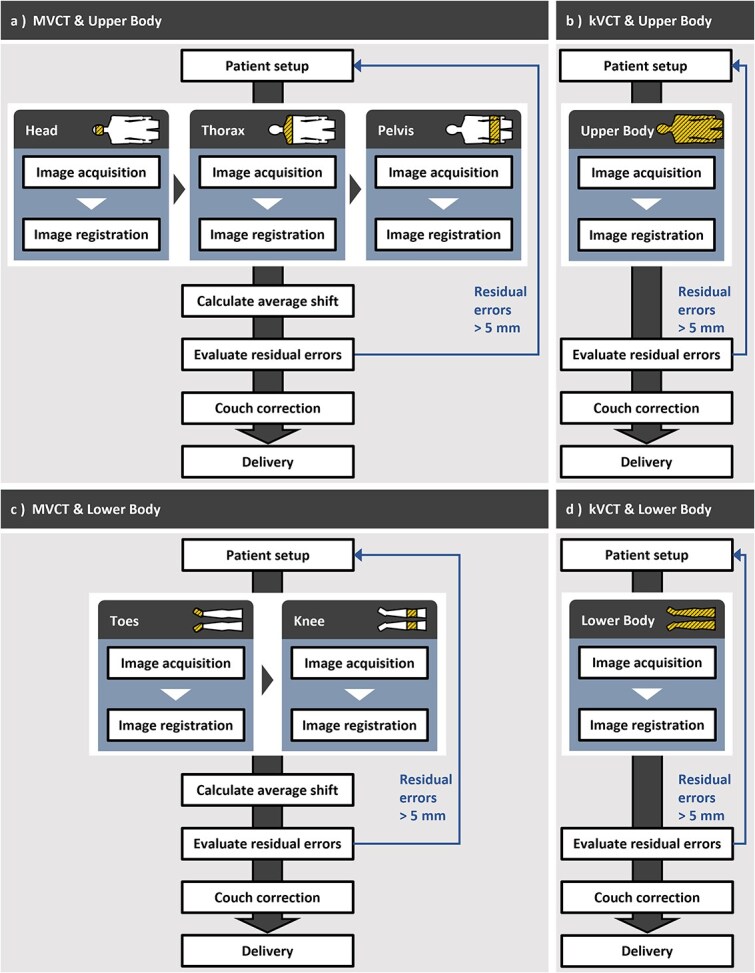
Flowchart of patient setup verification. Hatched regions indicate the image acquisition range.

Figures 2 and 3 illustrate the image acquisition range and patient setup verification protocol used at our hospital, respectively. The MVCT protocol used a slice thickness of 6 mm and an FOV of 390 mm, whereas the kVCT protocol used a slice thickness of 3.6 mm and an FOV of 500 mm. MVCT imaging was limited to specific anatomical regions, including the head, thorax, pelvis, knees, and toes. For each MVCT scan, automatic registration was initially performed, followed by manual fine-tuning for each anatomical region. The mean correction value was then computed and used for final couch correction. The residual errors were defined as the difference between each individual correction value and the mean correction value. Corrections were performed using the treatment couch whenever the residual error was within 5 mm. If the error exceeded 5 mm, a complete re-setup was necessary. For kVCT, a whole-body image registration approach was used to assess and correct the setup errors. The residual errors were visually evaluated for each slice by creating a 5 mm expanded region of interest (ROI) of the body and checking whether the body was contained within the ROI.

### Evaluation of the process duration and residual setup errors

This study recorded the time required for patient setup, image acquisition, image registration, re-setup (comprising the second setup, image acquisition, and image registration), and beam delivery in MVCT- and kVCT-guided TBI treatments. For MVCT, image acquisition and registration times represent the cumulative duration of multiple scans performed for distinct anatomical regions, including the head, thorax, pelvis, knees, and toes. In addition, to compare image-guidance strategies, we evaluated three workflows: (1) partial-body MVCT with region-specific registration, (2) whole-body kVCT with single-scan visual assessment, and (3) a modeled whole-body MVCT workflow constructed by extrapolating MVCT imaging time to match the kVCT scan range and assuming equivalent registration time. The analysis included data from 40 treatment fractions. Additionally, the residual setup errors along the lateral, longitudinal, and vertical axes were quantified using 100 region-specific MVCT datasets (20 each for the head, thorax, pelvis, knees, and toes) and 40 kVCT datasets (20 for UB and 20 for LB). The MVCT-based residual setup errors were directly extracted from clinical logs that included per-region corrections and calculated average shifts. In contrast, for kVCT, regional corrections were retrospectively derived and compared with the global shift applied during treatment to determine residual errors. A statistical analysis, including unpaired t-tests, was performed to identify significant differences between the two methods. Additionally, we defined the ‘residual pre-treatment setup error’ as the remaining discrepancy after the final couch correction prior to irradiation because no post-treatment imaging was conducted in this study. The t-tests were two-sided, with the statistical significance set at *P* < 0.05.

## RESULTS

### Efficiency of the first positioning cycle

**Figure 4:**
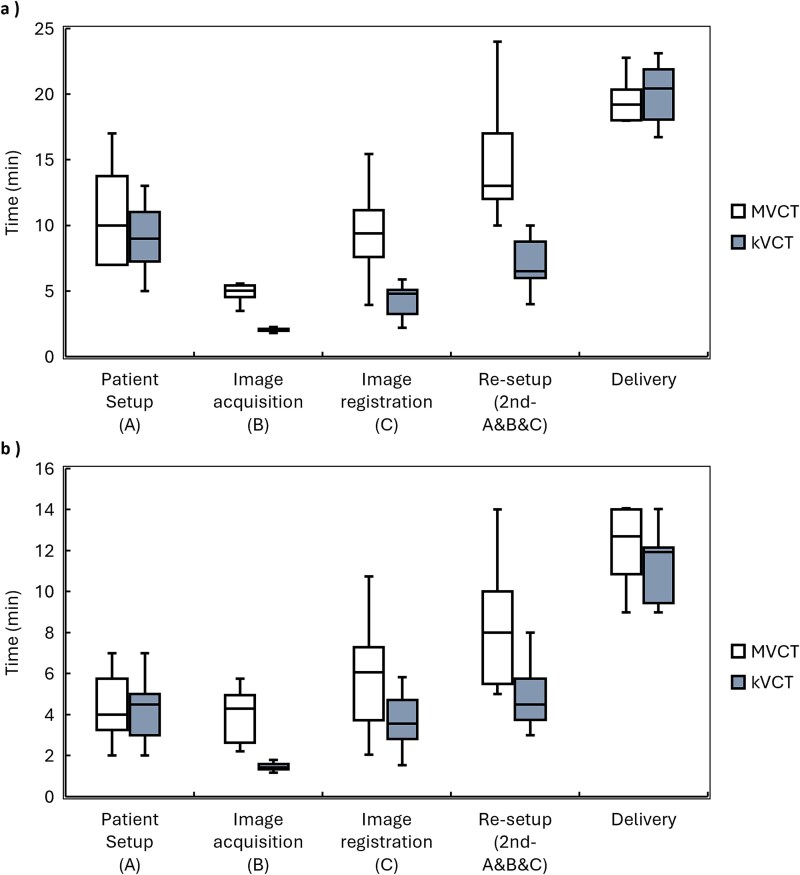
Times required for patient setup, image acquisition, image registration, re-setup (comprising the second setup, image acquisition, and image registration), and delivery. (a) Upper body. (b) Lower body.

Figure 4 and Table 2 present the times for patient setup (step A), image acquisition (step B), image registration (step C), re-setup, and delivery. For UB, the mean first-cycle duration of the combined A + B + C sequence was 15.9 ± 3.3 min with kVCT and 25.4 ± 4.6 min with MVCT. This difference stemmed almost entirely from the imaging steps: image acquisition was 61% faster with kVCT than with MVCT (*P* < 0.001), and image registration was 54% faster (*P* < 0.001), while patient setup times showed no statistically significant difference (*P* = 0.266). For LB, the first-cycle duration decreased from 14.45 ± 3.8 min with MVCT to 9.35 ± 2.4 min with kVCT. Once again, the improvement was primarily driven by faster image acquisition (*P* < 0.001) and registration (*P* = 0.003), with no significant difference observed in patient setup time (*P* = 0.589).

**Table 2:** Mean workflow times for MVCT and kVCT (min, mean ± SD)

Process step	Upper body			Lower body		
	MVCT	kVCT	*P*-value	MVCT	kVCT	*P*-value
Patient setup (A)	10.65 ± 3.3	9.50 ± 3.1	0.266	4.40 ± 1.3	4.15 ± 1.6	0.588
Image acquisition (B)	5.21 ± 1.5	2.02 ± 0.1	<0.001	4.05 ± 1.2	1.46 ± 0.2	<0.001
Image registration (C)	9.49 ± 2.9	4.38 ± 1.1	<0.001	6.00 ± 3.0	3.74 ± 1.3	0.003
Re-setup (2nd_A&B&C)	14.29 ± 5.0	7.25 ± 2.8	<0.001	8.15 ± 2.7	4.83 ± 1.7	0.013
Delivery	19.76 ± 1.7	20.01 ± 2.4	0.709	12.27 ± 1.6	11.47 ± 1.8	0.145

### Overall treatment times and impact of the second positioning cycle

The mean delivery times did not significantly differ between modalities (UB: 20.01 min with kVCT vs. 19.76 min with MVCT, *P* = 0.709; LB: 11.47 min with kVCT vs. 12.27 min with MVCT, *P* = 0.145). Consequently, the total duration of a complete UB and LB fraction—including the initial setup cycle and delivery—was 56.72 ± 5.3 min with kVCT and 71.82 ± 7.5 min with MVCT. When translational residual errors exceeded 5 mm, a second A + B + C cycle was initiated. Among the analyzed fractions, 30 of 40 MVCT-guided fractions and 18 of 40 kVCT-guided fractions required this second setup cycle. Notably, the kVCT cohort was treated later in the study period, which may have contributed to increased operator efficiency.

The additional time required for this second cycle averaged 7.25 ± 2.8 min for UB and 4.83 ± 1.7 min for LB with kVCT, compared to 14.29 ± 5.0 min for UB and 8.15 ± 2.7 min for LB with MVCT (UB, *P* < 0.001; LB, *P* = 0.013). Thus, kVCT enabled completion of residual error correction in approximately half the time required by MVCT.

### Protocol-wide comparison of the imaging workload

**Figure 5:**
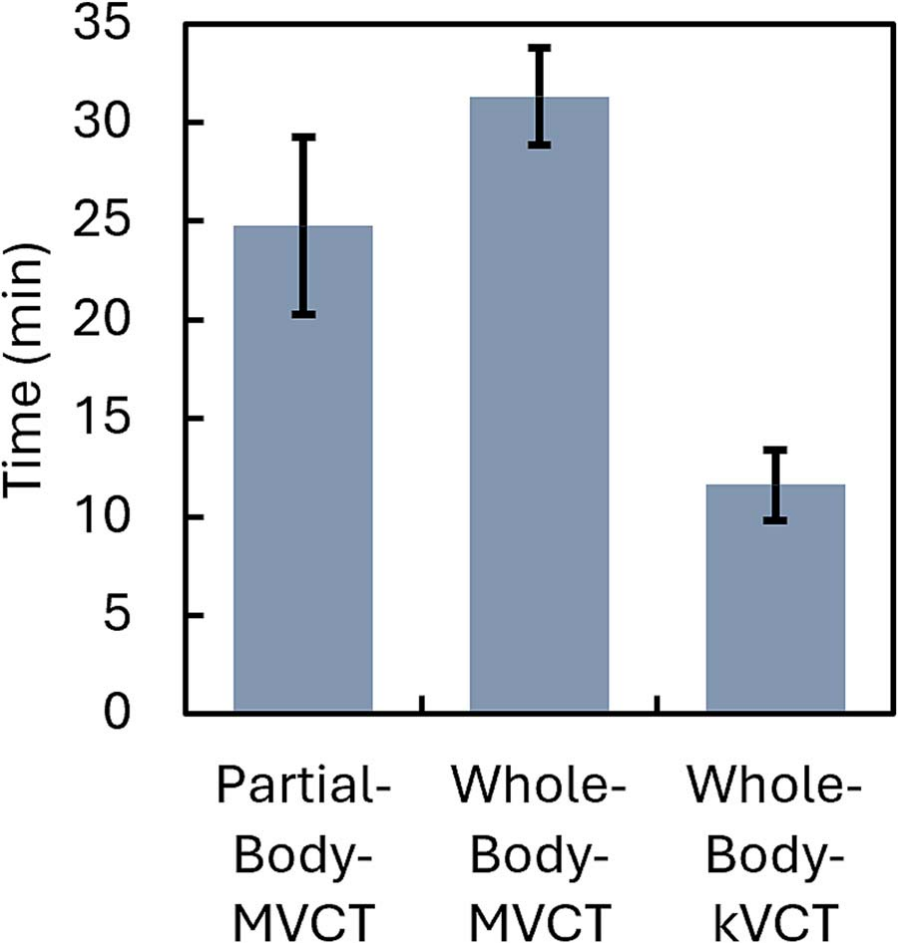
Cumulative imaging workload, defined as the sum of the acquisition and registration times for both the UB and LB fractions.

Figure 5 presents the cumulative imaging workload, defined as the sum of acquisition and registration times for both UB and LB fractions. The delivered partial-body MVCT workflow required 24.75 ± 4.5 min. To enable a direct comparison of the efficiency attributable to the imaging modality, we modeled a whole-body MVCT workflow covering the same craniocaudal range as kVCT. In this modeled scenario, to isolate the effect of the scan method, the image registration time was assumed equal to that of kVCT. This modeled ‘whole-body MVCT’ workflow required 31.34 ± 2.5 min. In contrast, the whole-body kVCT required only 11.60 ± 1.8 min. A one-way ANOVA confirmed a highly significant difference among the three strategies (*P* < 0.001), with whole-body kVCT being 2–3 times faster than either MVCT approach.

### Residual setup accuracy

**Figure 6:**
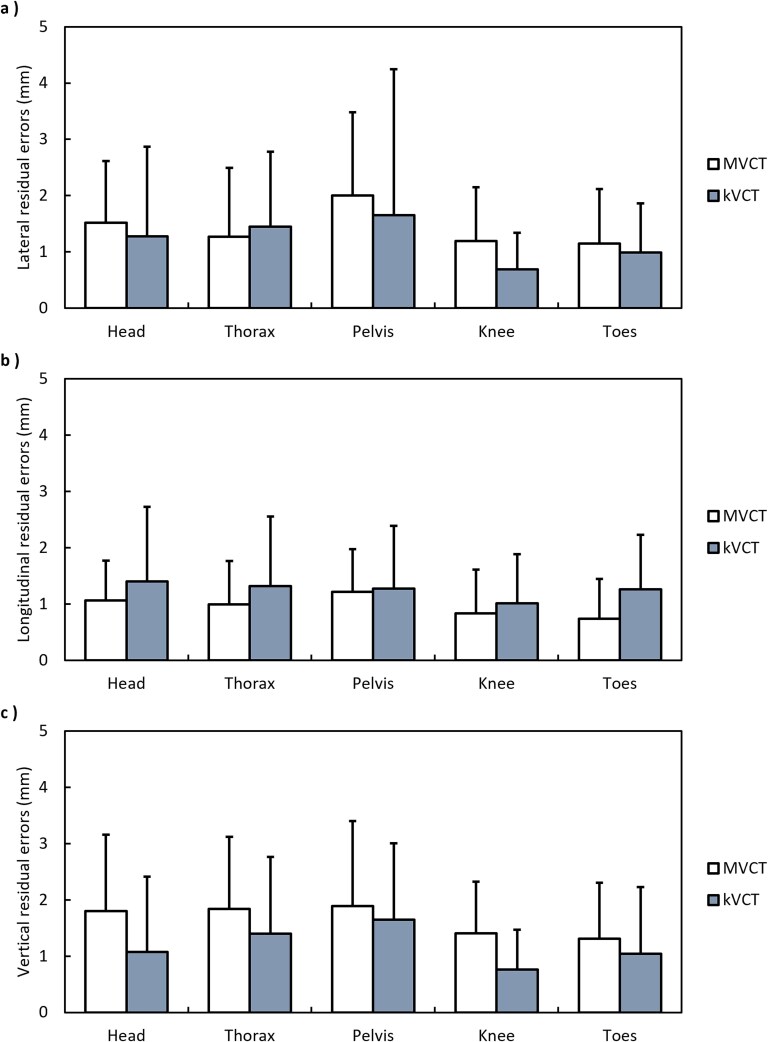
Mean ± SD absolute residual setup errors in the (a) lateral, (b) longitudinal, and (c) vertical directions of the head, thorax, pelvis, knees, and toes.

**Figure 7:**
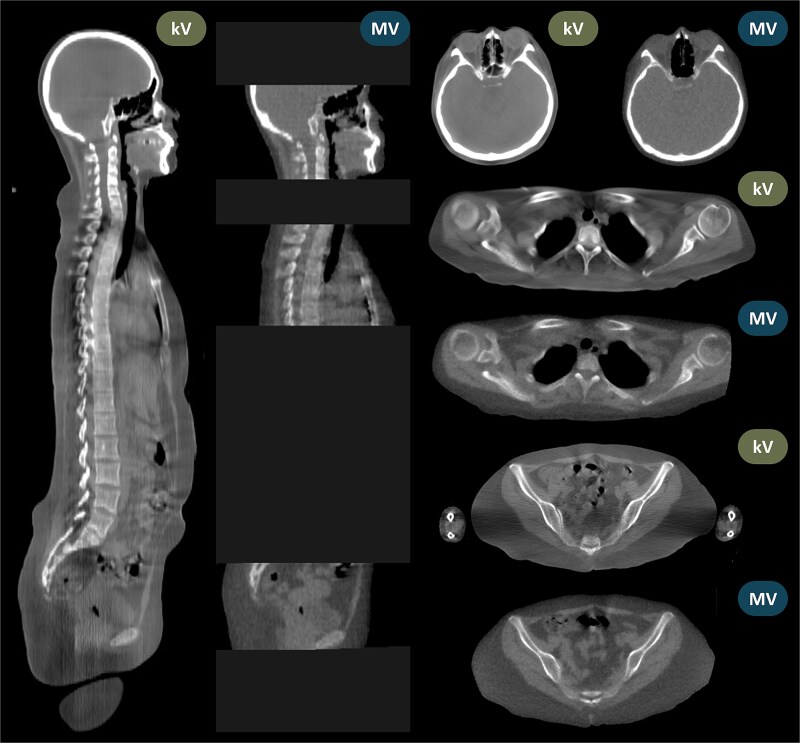
Axial and sagittal views of the same patient.

Figure 6 shows the mean ± SD absolute residual setup errors for the head, thorax, pelvis, knees, and toes along the lateral, longitudinal, and vertical directions. The mean ± SD absolute residual setup errors in the lateral, longitudinal, and vertical directions were 1.43 ± 1.18, 0.97 ± 0.75, and 1.65 ± 1.23 mm, respectively, with MVCT and 1.21 ± 1.57, 1.25 ± 1.10, and 1.19 ± 1.23 mm, respectively, with kVCT. All mean values and 95th-percentile measurements remained within the 5 mm tolerance recommended for helical tomotherapy-based TBI. Figure 7 shows representative axial and sagittal views of the same patient. The 500 mm FOV of kVCT captures the lower thoracic spine, lumbar spine, and arms—regions truncated in the 390 mm MVCT scan—and provides visibly superior soft-tissue contrast.

**Figure 8:**
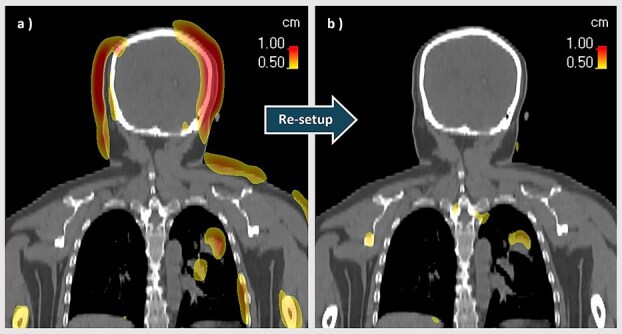
Results of deformable image registration from the fraction with the largest observed setup error. (a) Uncorrected kVCT for planning CT. (b) Re-setup of kVCT for planning CT. The color map represents the amount of deformation.

The benefit of the two-step verification strategy is illustrated in Fig. 8 for the case with the largest observed setup discrepancy among all analyzed fractions. Figure 8a shows the deformable image registration (DIR) between the planning CT and the kVCT image acquired after the first positioning cycle. Pronounced anatomical misalignments—including head displacement, shoulder elevation, and rib rotation—are apparent. After a complete re-setup, including patient repositioning and a second kVCT scan (Fig. 8b), these misalignments were substantially corrected. Notably, DIR was used solely for qualitative visualization in this figure. All clinical corrections were implemented via translational couch shifts. Rotational and complex anatomical deviations were addressed through manual patient repositioning, rather than deformable or rotational corrections.

## DISCUSSION

To our knowledge, this technical report presents the first quantitative comparison of three image-guidance strategies for helical tomotherapy-based TBI: (1) partial-body MVCT, commonly used in current clinical workflows; (2) whole-body kVCT, recently implemented at our institution; and (3) a modeled whole-body MVCT workflow matched to the craniocaudal scan range of kVCT. By isolating the effects of imaging modality and scan coverage, this study provides practical data to inform evidence-based optimization of setup protocols in the highly time-constrained context of TBI.

Our analysis of 40 treatment fractions across 14 patients revealed that kVCT significantly reduced the duration of all image-dependent processes without compromising pre-treatment setup accuracy. A key contributor to this efficiency was the accelerated acquisition speed of kVCT, which allowed continuous scanning over extended craniocaudal ranges and eliminated the need for multiple segmented scans inherent to the MVCT workflow. In UB setups, kVCT reduced image acquisition time by 61% and registration time by 54% (Section 3.1). For an entire treatment fraction—including the initial positioning cycle and beam delivery—the average duration was 56.7 min with kVCT compared to 71.8 min with the partial-body MVCT workflow (Section 3.2). When a second positioning cycle was required, the extra imaging time with kVCT remained approximately half that of MVCT (UB: 7.3 vs. 14.3 min; LB: 4.8 vs. 8.2 min).

To enable a direct comparison, we simulated a whole-body MVCT workflow with a continuous scan covering the same craniocaudal range as kVCT. For a conservative estimate, the registration time from the kVCT dataset was applied to the simulated MVCT protocol. Even under these optimized conditions, the cumulative imaging workload for whole-body MVCT was 31.3 min—nearly three times longer than the 11.6 min recorded for kVCT (Section 3.3). This pronounced discrepancy highlights the intrinsic efficiency of fan-beam kVCT in whole-body imaging workflows.

Despite the shorter scan duration, kVCT maintained mean absolute residual setup errors below 2 mm across all axes and anatomical regions, with all 95th-percentile values remaining within the 5 mm tolerance recommended for tomotherapy-based TBI [18, 19]. In this study, residual setup error was defined as the discrepancy persisting after all couch corrections and immediately prior to treatment delivery. As no post-treatment imaging was performed, these values do not account for intrafractional motion and therefore reflect pre-treatment positioning accuracy rather than full geometric fidelity during irradiation. While comparative dose data are limited, prior reports indicate that kVCT generally delivers a lower imaging dose than MVCT [15]. Given the relatively infrequent imaging required in TBI protocols, the clinical impact of this dose difference is likely minimal.

These findings have significant clinical implications. Prolonged treatment durations are associated with increased patient discomfort and a heightened risk of intrafractional motion, both of which can compromise dose accuracy [10]. Although this study did not directly measure patient motion during irradiation, the time savings achieved with kVCT can facilitate the incorporation of advanced planning strategies. For example, reducing the helical pitch improves dose uniformity by mitigating thread effects—particularly in larger patients—but is typically associated with increased beam-on time [20]. By decreasing the time required for image acquisition and registration, kVCT may enable such dose-optimization techniques to be applied without extending the overall treatment duration.

In high-throughput facilities such as ours, where more than 30 patients are treated daily using the Radixact system, the reduced setup time also contributes to improved schedule adherence, decreased patient waiting times, and lower staff overtime. While these operational advantages were not directly quantified, they are especially pertinent in tightly scheduled regimens, such as 12 Gy protocols delivered in twice-daily fractions.

From a patient-centered perspective, shorter treatment durations may help reduce both physical and psychological burdens. One such burden is the discomfort associated with thermoplastic immobilization devices. While these devices are indispensable for high-dose regimens, their necessity may warrant re-evaluation in uniform low-dose protocols, such as the 4 Gy regimen. Notably, tomotherapy delivers radiation in a head-to-foot sequence, meaning the cranial region is irradiated early in the session. Given that the risk of intrafractional motion increases with time, the head may be inherently less susceptible to motion than regions treated later. This temporal aspect supports the hypothesis that head immobilization could be selectively omitted in low-dose scenarios where strict positional reproducibility is less critical. Such an approach may be particularly beneficial for patients who are unable to tolerate thermoplastic masks, including those with claustrophobia. Nevertheless, any relaxation of immobilization protocols must be approached with caution and rigorously validated through prospective clinical studies.

This study has several limitations. First, its retrospective design and relatively small sample size limit the generalizability of the findings. Second, the residual setup errors assessed in this study do not account for intrafractional motion, which remains an important subject for future research. Incorporating post-treatment kVCT imaging in subsequent protocols can provide more comprehensive assessments of geometric accuracy. Finally, although this report focuses on pre-treatment setup accuracy, the integration of kVCT into adaptive TBI strategies represents a promising avenue for future development.

In conclusion, our results demonstrate that kVCT offers significant advantages over MVCT for TBI using helical tomotherapy, including rapid whole-body imaging, an expanded FOV, and a substantial reduction in overall treatment time. These workflow improvements not only preserve but also enhance setup accuracy by enabling a comprehensive, single-scan evaluation of the entire body. These advantages highlight the potential of kVCT to optimize TBI protocols, improve patient comfort, and streamline the delivery of complex radiation therapy.
